# ﻿Complete mitochondrial genome and phylogenetic analysis of the Atrato slider, *Trachemysmedemi* (Testudines, Emydidae)

**DOI:** 10.3897/zookeys.1224.136863

**Published:** 2025-01-31

**Authors:** Sebastián Cuadrado-Ríos, Mario Vargas-Ramírez, Christian Kehlmaier, Uwe Fritz

**Affiliations:** 1 Grupo de Biodiversidad y Conservación Genética, Instituto de Genética, Universidad Nacional de Colombia, Bogotá, Colombia Universidad Nacional de Colombia Bogotá Colombia; 2 Museum of Zoology, Senckenberg Natural History Collections Dresden, 01109 Dresden, Germany Museum of Zoology, Senckenberg Natural History Collections Dresden Dresden Germany

**Keywords:** Chocó, Colombia, mitogenome, phylogeny, primer walking, turtle

## Abstract

The mitochondrial genome of three *Trachemysmedemi* was sequenced and annotated for the first time. The mitochondrial genome is a circular DNA molecule of 16,711–16,810 bp in size, with 60.9% AT content. It includes 13 protein-coding genes, two rRNA genes, 22 tRNA genes, and the non-coding control region. The genome composition is characterized by a positive AT skew (0.123) and a negative GC skew (-0.342). Phylogenetic analyses based on complete mitogenomes, which lack some *Trachemys* species, placed *T.medemi* as sister to *T.venusta*. Phylogenies from the same dataset, but including available shorter mtDNA information for most *Trachemys* species, recovered *T.medemi* as sister to *T.dorbigni*, and this clade was sister to *T.venusta*, *T.yaquia*, and *T.ornata*. The newly obtained data are valuable for future mitogenomic investigations on *Trachemys*. Furthermore, our results underline the impact of incomplete taxon sampling.

## ﻿Introduction

The Atrato slider (*Trachemysmedemi* Vargas-Ramírez, del Valle, Ceballos & Fritz, 2017) is a species of freshwater turtle with a narrow distribution. It is restricted to the Atrato Basin of northwestern Colombia (Vargas-Ramírez et al. 2017; TTWG 2021) and one of the four endemic turtle species of the country (Páez et al. 2022). Only described in 2017, *Trachemys* individuals from the Atrato Basin were previously misidentified as various other taxa, including *Trachemysvenustauhrigi* McCord, Joseph-Ouni & Blanck, 2010, a Central American taxon (TTWG 2017, 2021; see also the review in Vargas-Ramírez et al. 2017). Other slider turtle populations in Colombia and Venezuela actually represent *T.venusta* (Gray, 1856), the species with which the Atrato sliders were associated before. However, phylogenetic analyses using mitochondrial and nuclear DNA sequences (Vargas-Ramírez et al. 2017; Fritz et al. 2023, 2024) recovered *T.medemi* as sister to *T.dorbigni* (Duméril & Bibron, 1835), a species which occurs far away in Brazil, Uruguay and Argentina. To expand our knowledge of *T.medemi*, we sequenced its entire mitogenome for the present study. In the context of taxonomic uncertainties and introgression in *Trachemys* (Fritz et al. 2023, 2024), these data can be critical for further taxonomic and phylogenetic studies, and also provide a valuable resource for mitogenomic studies in turtles.

## ﻿Material and methods

We used ethanol-preserved blood samples from three *Trachemysmedemi* from the Banco de Tejidos de la Biodiversidad Colombiana (BTBC), Instituto de Genética, Universidad Nacional de Colombia, Bogotá. To maximize the utility of these data for future analyses, we selected three individuals from different localities (BTBC12643: between Las Brisas and Llano Rico, Riosucio, Chocó, 7.3267, -76.8129; BTBC13199: Reserva Natural Surikí, Turbo, Antioquia, 7.7704, -76.8838; BTBC13207: Ciénaga de Napipí, Bojayá, Chocó, 6.6277, -76.9489).

DNA was extracted using the innuPREP DNA Mini Kit 2.0 (Analytik Jena), with a final elution of 80 µl milliQ water. The complete mitochondrial genome was amplified using long-range PCR followed by primer-walking procedures. Using primers from Fritz et al. (2012, 2023), two long-range PCR reactions (LR1 and LR2) were performed, yielding amplicons with lengths of 11,824 bp and 6797 bp, which overlap by more than 1000 bp. Long-range PCR products were purified using the ExoSAP-IT enzymatic clean-up (USB Europe, Staufen, Germany). These amplicons cover most of the mitochondrial genome, except for the first part of the tRNA-Phe and the final 3’-end of the control region. These regions were amplified using standard PCR procedures and custom primers designed from the consensus sequence of the primer-walking PCR products. The cleaned products were Sanger sequenced using a set of primers compatible with the mitochondrial genome of *Trachemysscriptaelegans* (GenBank accession number MW019443; Suppl. material 1: table S1). The initial batch of sequences was mapped onto the same mitogenome sequence from *T.s.elegans* (MW019443) and curated using GENEIOUS R7 (http://geneious.com). From this assembly, new sequencing primers were designed to close the gaps between the sequences. This process was repeated twice, resulting in a total of 14 newly designed primers (Suppl. material 1: table S1). Detailed laboratory procedures are provided in the Suppl. material. Mitogenomes were annotated with MITOS v. 2.1.7 (Bernt et al. 2013) through the Proksee server (Grant et al. 2023; Suppl. material 1: table S2). Initiation and termination codons were identified using ORFFINDER (https://www.ncbi.nlm.nih.gov/orffinder). AT skew and GC skew were calculated to describe the base composition (Perna and Kocher 1995). The new mitogenomes have been deposited in the European Nucleotide Archive (ENA) under accession numbers OZ183365–OZ183367.

Phylogenetic analyses using maximum likelihood (ML) and Bayesian inference (BI) were performed with a complete mitogenome dataset, which included all available complete mitogenomes for *Trachemys* (Suppl. material 1: table S3). However, no mitogenomes have been published for the two taxa (*T.d.dorbigni*, *T.d.adiutrix*) previously identified as sister to *T.medemi* (Vargas-Ramírez et al. 2017; Fritz et al. 2023, 2024). Therefore, we re-analyzed the mitogenomes from our initial dataset together with the 3221-bp-long mtDNA dataset from Fritz et al. (2024), with missing information coded as Ns. For further details, see Suppl. material 1.

## ﻿Results

We obtained three complete mitogenomes of *Trachemysmedemi*, ranging from 16,711 bp to 16,810 bp. The length variation was due to the absence of a 92-bp-long repetitive sequence in the control region of one mitogenome (ENA accession number OZ183367) and some minor indels in the terminal repetitive part of the control region.

**Figure 1.**
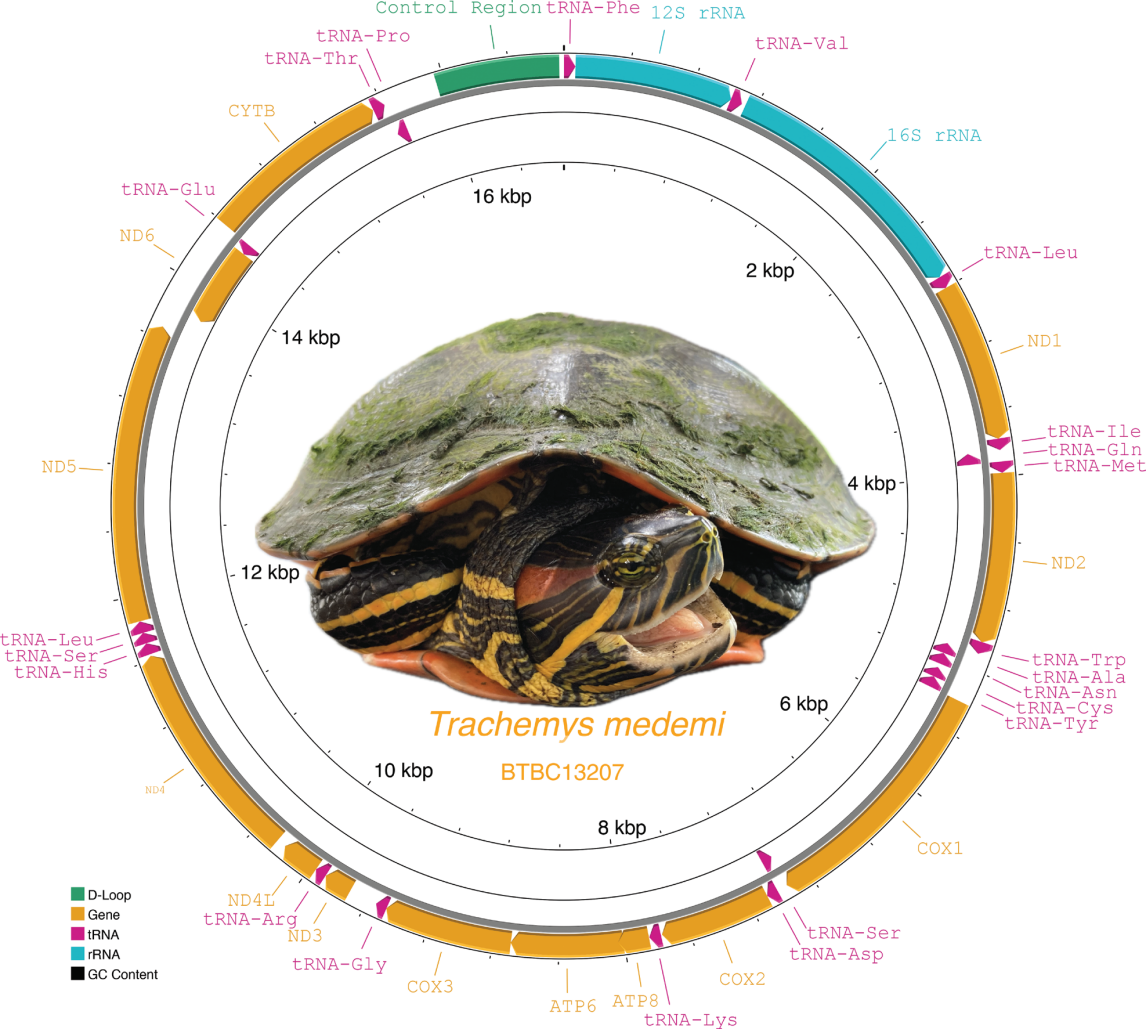
Circular view of the complete annotated mitogenome of the Atrato slider (*Trachemysmedemi*), displaying 13 protein-coding genes, two rRNA genes, 22 tRNA genes, and the control region (ENA accession number OZ183365). Inset photo: *T.medemi*, adult female from Ciénaga de Napipí, Bojayá, Chocó, Colombia. Photo: Sebastián Cuadrado-Ríos.

**Figure 2.**
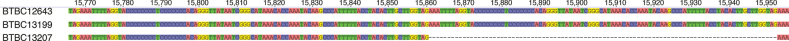
Part of the alignment of the three *Trachemysmedemi* mitogenomes obtained in the present study illustrating the absence of a 92-bp-long duplicated sequence in the control region of BTBC13207, spanning from position 15,861 to 15,952.

The mitogenome consists of 13 protein-coding genes, 2 ribosomal RNA (rRNA) genes, 22 transfer RNA (tRNA) genes and the non-coding control region (Fig. 1, Suppl. material 1: table S2), as in other *Trachemys* and related emydid species (Fritz et al. 2023, 2024; Ren et al. 2024). Nine of the 37 genes are encoded on the light strand; the remaining genes are on the heavy strand. The three mitogenomes differ in two mutations in the ND1 gene, one mutation in the ND2 gene, two mutations in the ND4L gene, two mutations in the ND4 gene, one mutation in the ND5 gene, one mutation in the ND6 gene, and one mutation and the absence/presence of a duplicated part of the control region (92 bp) and a few minor indels in the terminal repetitive part of the control region (Fig. 2). The genome composition is A: 34.3%, C: 26.1%, G: 12.8%, T: 26.6%, with a slight AT bias (60.9%), a positive AT skew (0.123), and a negative GC skew (-0.342). The AT skew falls within the range of other *Trachemys* species, but is higher than in most previously sequenced emydid mitogenomes. The GC skew is also higher than in most of the other emydid mitogenomes (Ren et al. 2024). As in other *Trachemys* species, the GC content was consistent across the mitogenome, ranging from 38.1% to 39.5%, except for the control region with a GC content of 32.7% (Suppl. material 1: table S3).

Of the 22 tRNA genes in the mitogenome of *T.medemi*, 14 are encoded on the heavy strand and eight on the light strand (Fig. 1). The tRNA genes ranged between 67 and 75 bp in length and exhibited a positive AT skew (0.128) and A+T bias (61.7%; Suppl. material 1: table S3). The 12S rRNA gene is located between the initial tRNA-Phe and tRNA-Val; the 16S rRNA is between tRNA-Val and tRNA-Leu (Fig. 1). The rRNA genes are 976 bp and 1635 bp long, respectively, and show an A+T bias (60.2%), a positive AT skew (0.282) and a negative GC skew (-0.161; Suppl. material 1: table S3). The control region is located between the tRNA-Pro and tRNA-Phe genes. In one mitogenome (BTBC13207), the control region is distinctly shorter due to the absence of a repetitive sequence of 92 bp length (Fig. 2). The control region has only a very slightly positive AT skew (0.002) and a negative GC skew (-0.266). The mitogenomic characteristics of *T.medemi* and other species are detailed in Suppl. material 1: table S3.

**Figure 3.**
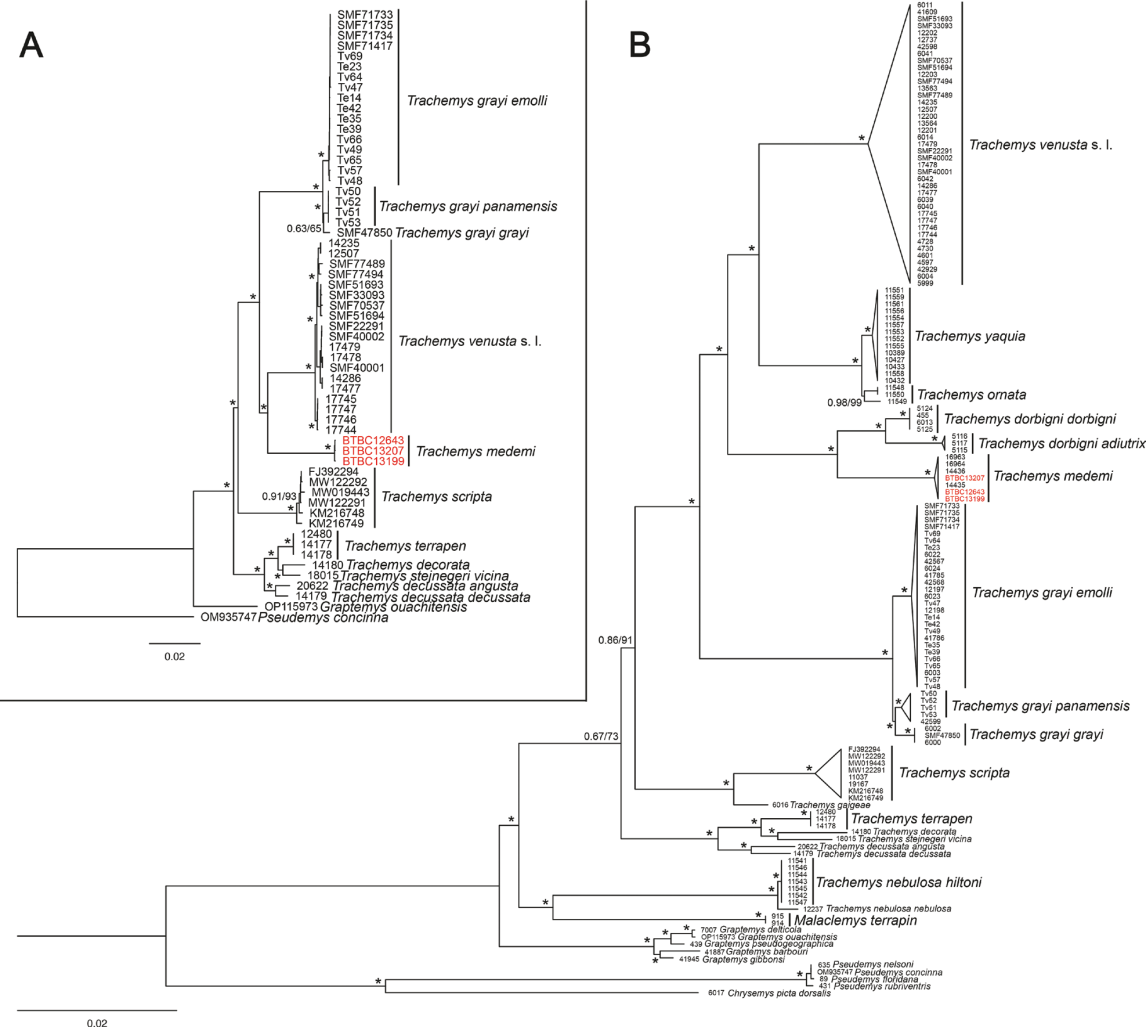
Maximum likelihood trees estimated with (**A**) mitogenomes and (**B**) mitogenomes combined with data from Fritz et al. (2024), representing nearly all *Trachemys* taxa. Outgroup (*Deirochelysreticularia*) removed for clarity in (**B**). The newly sequenced *T.medemi* mitogenomes are highlighted in red. Numbers at nodes represent posterior probabilities from a Bayesian phylogeny (left) and bootstrap values from the ML tree (right). Asterisks indicate maximum support from both approaches.

Phylogenetic analyses using complete mitogenomes resulted in similar topologies with robust support for the major clades (Fig. 3A). Both analyses placed *T.medemi* as sister to *T.venusta* sensu lato, with both species forming a clade that is sister to *T.grayi*. When the mitogenomes were aligned with the 3221-bp-long mtDNA dataset from Fritz et al. (2024), the resulting phylogenies showed similar topologies, also with robust support for the main clades (Fig. 3B). However, these phylogenies placed *T.medemi* as sister species to *T.dorbigni*, with this clade being sister to a clade composed of *T.venusta* sensu lato, *T.yaquia*, and *T.ornata* (Fig. 3B). The phylogenetic position of the West Indian species *T.terrapen*, *T.decorata*, *T.stejnegeri*, and *T.decussata* was weakly resolved (Fig. 3B).

## ﻿Discussion and conclusion

In this study, we sequenced, assembled and characterized three mitogenomes of *Trachemysmedemi*, representing the first complete mitogenomes for the species. One mitogenome (BTBC13207) lacks a duplicated sequence between positions 15,861 and 15,952 (Fig. 2) in the right domain of the control region according to Bernacki and Kilpatrick (2020). The turtle was captured in the Ciénaga de Napipí, Bojayá, Chocó, Colombia, at the southernmost edge of the distribution range (see Vargas-Ramírez et al. 2017). Furthermore, an examination of the mitogenome alignment revealed that this phenomenon also occurs in *T.venusta*: the only mitogenome published for *T.v.iversoni* (OZ038161), which does not significantly differ from the mitogenomes of *T.v.venusta* and *T.v.uhrigi* (Fritz et al. 2024), misses the same duplicated region as BTBC13207. It remains to be tested whether this has population genetic implications or merely represents individual variation.

Our phylogenetic analyses based on complete mitogenomes (Fig. 3A) recovered *T.medemi* as sister to *T.venusta* sensu lato in a maximally supported clade. However, when additional mtDNA sequences were included, *T.medemi* was sister to *T.dorbigni*, with these two species together representing the sister clade of another clade composed of *T.venusta* sensu lato, *T.yaquia*, and *T.ornata* (Fig. 3B). Thus, our results underline the impact of incomplete taxon sampling when the mitogenome data are considered alone and confirm previous phylogenetic analyses based on shorter mtDNA sequences (Vargas-Ramírez et al. 2017; Fritz et al. 2023, 2024). Furthermore, the mitochondrial genomes of the narrow-endemic *T.medemi* are a valuable resource for future mitogenomic investigations.

## ﻿Additional information

### Author ORCIDs

Sebastián Cuadrado-Ríos https://orcid.org/0000-0002-1060-820X

Mario Vargas-Ramírez https://orcid.org/0000-0001-8974-3430

Christian Kehlmaier https://orcid.org/0000-0001-9622-0566

Uwe Fritz https://orcid.org/0000-0002-6740-7214

### Data availability

The mitochondrial genome sequences are openly available from the European Nucleotide Archive (ENA) at https://www.ebi.ac.uk/ena/browser/home under the accession numbers OZ183365-OZ183367 (BioProject number PRJEB79932).

## Supplementary materials

Supplementary material 1 Supplementary information Data type pdf Explanation note Details for long-range PCR, primer walking and phylogenetic analyses. https://binary.pensoft.net/file/1239700This dataset is made available under the Open Database License (http://opendatacommons.org/licenses/odbl/1.0/). The Open Database License (ODbL) is a license agreement intended to allow users to freely share, modify, and use this Dataset while maintaining this same freedom for others, provided that the original source and author(s) are credited.Sebastián Cuadrado-Ríos, Mario Vargas-Ramírez, Christian Kehlmaier, Uwe Fritz
