# Supplementary material for: ﻿Complete mitochondrial genome and phylogenetic analysis of the Atrato slider, Trachemysmedemi (Testudines, Emydidae)
Source: Zookeys. 2025 Jan 31;1224:253–60. doi: 10.3897/zookeys.1224.136863 (PMC11806343; doi:10.3897/zookeys.1224.136863)
Supplement: Supplementary material 1 — Supplementary information [file zookeys-1224-253_article-136863__-s001.pdf]

Complete mitochondrial genome and phylogenetic analysis of the Atrato slider, *Trachemys medemi* (Testudines: Emydidae)

Sebastián Cuadrado-Ríos[1,2], Mario Vargas-Ramírez[1,2], Christian Kehlmaier[2], Uwe Fritz[2]

1 *Grupo de Biodiversidad y Conservación Genética, Instituto de Genética, Universidad Nacional de Colombia, Bogotá, Colombia*

2 *Museum of Zoology, Senckenberg Natural History Collections Dresden, 01109 Dresden, Germany*

Supplementary information

Long-range PCR and primer walking. Two long-range PCR reactions were performed using DNA extractions from three *Trachemys medemi* samples to target the mitogenome. The resulting amplicons overlap by more than 1000 bp and have a length of 11,824 bp and 6797 bp, respectively. Following the methodology of Fritz et al. (2012, 2023), the following primer pairs were employed for the long-range PCR reactions: Tscripta_tPhe.for + Tscripta_tLeu_a.rev and Tscripta_tArg_b.for + DES2_rev. Each reaction was performed in a 50 µl volume containing 1 unit of TaKaRa LA Taq DNA polymerase, Hot-Start Version (Clontech Laboratories Inc.), 10 mM of dNTPs, 5 pmol of each primer, and volumes according to manufacturer's recommendation. PCR conditions included an initial denaturation at 93°C for 3 min, 35 cycles of 93°C for 20 s, 57°C for 30s, 68°C for 12 min, and a final elongation step at 68°C for 20 min. PCR products were visualized in a 2% agarose gel and purified using the ExoSAP-IT enzymatic clean-up (USB Europe, Staufen, Germany). Cleaned long-range PCR products were then used for PCR sequencing reactions. Initially, amplification and sequencing were performed using primers specified in the Supplementary Table 1. Subsequently, specific primers designed from the *T. medemi* sequences were used to fill the remaining gaps in the assembly. The first part of the tRNA-Phe and the final of control region's 3'-end were amplified using the primers medemitRNA-Phe (5'-TGGGTAACCAATATACCCCA-3'), medemitRNA-Phe2 (5'-TTAGATTGCTAGGGC GTTTT-3'), and medemiCRend (5'-ACCCACGACAGTAATTTTCA-3') under the following thermocycling conditions: Initial denaturation at 94°C for 5 min, followed by 30 cycles of 94°C for 30s, 60°C for 45s, 72°C for 30 s, and final elongation at 72°C for 10 min. Sequencing was performed on an ABI 3730 Genetic Analyzer (Applied Biosystems, Foster City, CA, USA) using the BigDye Terminator v3.1 Cycle Sequencing Kit (Applied Biosystems) following manufacturer's instructions.

Phylogenetic analyses. Phylogenetic analyses were conducted using two datasets. The first dataset included *Trachemys* mitogenomes obtained from Russel and Beckenbach (2008), Yu et al. (2014), Park et al. (2021), Ryu et al. (2021), and Fritz et al. (2023, 2024), alongside with mitogenomes from *Pseudemys concinna* (OM935747; Park et al. 2022) and *Graptemys ouachitensis* (OP115973) as outgroups. Protein-coding genes were screened for internal stop codons in GENEIOUS R7 (http://geneious.com). Mitogenome alignment was performed with MUSCLE (Edgar 2004) and manually curated to correct alignment

errors. Problematic sequence features, such as internal stop codons in coding genes, genes or tRNAs overlapping regions, frame shift inducing in coding region, and non-coding spacer DNA were identified and 232 positions were removed from the alignment. For the second dataset, our dataset was concatenated with the mtDNA alignment used by Fritz et al. (2024) covering almost all continental *Trachemys* taxa, except for *T. hartwegi*. Their dataset comprised 3221 bp and included sequences of the partial 12S gene and the complete ND4L, ND4, and cyt *b* genes plus part of the adjacent tRNA-Thr gene. Missing data was coded as Ns. The best partitioning schemes and evolutionary models (Supplementary Tables S4, S5) were determined by using a greedy search and the Bayesian Information Criterion in PARTITIONFINDER 2 (Lanfear et al. 2017). For the extended mtDNA dataset, PARTITIONFINDER 2 detected a single partitioning scheme with HKY+G+I model as the best fit. This partition scheme was used for the complete mitogenome dataset.

Phylogeny was analyzed using maximum likelihood (ML) and Bayesian inference (BA). ML analyses were performed in IQ-TREE 1.6.12 (Nguyen et al. 2015) with 1000 non-parametric thorough bootstrap replicates to assess node support, plotted against the best tree obtained. Bayesian analyses were conducted in MRBAYES 3.2.6 (Ronquist et al. 2012), with two simultaneous runs and four chains each, sampling every 1000th generation for 50 million generations. After a burn-in of 25%, convergence was assessed by verifying that the average standard deviation of split frequencies was below 0.01 and the effective sample size exceeded 200 for all parameters using TRACER 1.7.1 (Rambaut et al. 2018).

Supplementary Table S1. Primers used for PCR and sequencing. Position according to the mapping of each primer to the mitochondrial genome of *Trachemys scripta elegans* (GenBank accession number MW019443).

Primer	Sequence (5'-3')	LR-PCR	Position
Tscripta_tPhe.for	AGCACGGCACTGAAGTTGCC	1	22-41
L25195_for	AAACTGGGATTAGATACCCCACTAT	1	506-530
sulcata_cytb-12S_Rev	AGGCAAGTCGTAACAAGG	1	1001-1018
16SA_For	CGCCTGTTTATCAAAAACAT	1	1985-2004
graeca_cytb_16S_Rev	AACCGTGCAAAGGTAGCGTA	1	2078-2097
16SB_Rev	ACGTGATCTGAGTTCAGACCGG	1	2570-2591
medemi1F*	AAATGCAAAAGGCCTAAACC	1	2760-2779
medemi5F*	ACTCTCAGCAGGATTCCTAT	1	3643-3662
medemi5R*	ACCAATTACACCAACCCTAC	1	5022-5041
L-turtCOI_For	ACTCAGCCATCTTACCTGTGATT	1	5420-5443
medemi1R*	TTTGGTCGTCTCCTAAAAGG	1	5552-5571
Jerry_For	CAACAYTTATTTTGATTTTTTGG	1	6130-6152
medemi2F*	TTTTAGGCTTCATCGTGTGA	1	6259-6278
H-turtCOIb_Rev	CGAGCCTATTTTACATCTGCAAC	1	6340-6362
medemi6F*	CATGTACAAACCCAAGAAAG	1	6966-6985
medemi2R*	TCTGAGCATTGTCCGTAAAA	1	7671-7690
medemi6R*	TTTTACGGACAATGCTCAGA	1	7695-7714
nigraATP6-cytb_For	AAGCCCACAAAYCCTWGGAA	1	8070-8090
graeca_12S_ATP6_Rev	ACAGCCAATTTAACAGCCGG	1	8525-8545
medemi3F*	CAGCAGCAGCACTACTTATA	1	8764-8783
nebulosa_long_F_For	CCTAGAATGAGCAGAATCGA	1	9911-9931
Tscripta_tArg_b.for	CTTAGTTAATCGTGATTAAACTCCACGGC	1, 2	9963-9992
medemi3R*	TAGGTGAGTTTGGTGTAACG	1, 2	10035-10054
L-ND4_For	GTAGAAGCCCCAATCGCAG	1, 2	10951-10971
Tscripta_tHis_a.for	CATTAGACTGTGGCTCTAAAAATAGGAGTT	1, 2	11692-11721
Tscripta_tHis_b.rev	GTGGCTCTAAAAATAGGAGTTCAAACCTC	1, 2	11701-11730
medemi4F*	GCTCCCTCACTTTTAAAGGA	1, 2	11777-11796
LR1-Rev_Tscripta_tLeu_a.rev	CCATTGGTTTTAGAAACCATCCACCC	1, 2	11828-11854
H-Leu_Rev	TGGTGCAAATCCAAGTAAAAGTAAT	2	11855-11880
medemi7F*	CTGCCCTATCCCAAAATGAT	2	12747-12766
medemi7R*	TTAACAACAACCCTCAACCA	2	13931-13950
nigra_ND6-12S_For	AACYAACATCCCRCCCAAAT	2	14199-14219
medemi4R*	TAGTCTTAGGAGCAGCTTCT	2	14227-14246
sulcata_cytb-12S_For	CCTACAAATCACCACCGGAA	2	14599-14618
sulcata_ATP6-cytb_Rev	AGCCCATATCACCCGAGATG	2	14671-14690
Tscripta_tThr_a.rev	AAAGCATTGTCTTGTAAACCAAAGACTGA	2	15635-15664

*Primers newly designed for this study.

Supplementary Table S2. Annotation of the *Trachemys medemi* mitogenome BTBC13207.

Name	Start	Stop	Length	Strand	Intergenic/overlapping nucleotides
tRNA-Phe	1	70	70	+	0
12S rRNA	71	1039	969	+	0
tRNA-Val	1040	1110	71	+	0
16S rRNA	1111	2726	1616	+	1
tRNA-Leu	2728	2802	75	+	0
ND1	2803	3773	971	+	0
tRNA-Ile	3774	3843	70	+	-1
tRNA-Gln	3843	3914	72	-	-2
tRNA-Met	3914	3982	69	+	-1
ND2	3983	5021	1039	+	0
tRNA-Trp	5022	5096	75	+	1
tRNA-Ala	5098	5166	69	-	1
tRNA-Asn	5168	5240	73	-	1
tRNA-Cys	5267	5332	66	-	-5
tRNA-Tyr	5333	5403	71	-	1
COX1	5405	6948	1544	+	-5
tRNA-Ser	6944	7015	72	-	2
tRNA-Asp	7018	7087	70	+	0
COX2	7088	7774	687	+	1
tRNA-Lys	7776	7847	72	+	-2
ATP8	7850	8038	189	+	-31
ATP6	8008	8691	684	+	-1
COX3	8691	9474	784	+	-1
tRNA-Gly	9475	9543	69	+	0
ND3	9544	9893	350	+	0
tRNA-Arg	9894	9963	70	+	0
ND4L	9964	10261	297	+	-7
ND4	10255	11635	1381	+	0
tRNA-His	11636	11705	70	+	0
tRNA-Ser	11706	11771	66	+	0
tRNA-Leu	11772	11843	72	+	-2
ND5	11842	13671	1830	+	128
ND6	13800	14324	525	-	0
tRNA-Glu	14325	14392	68	-	4
CYTB	14397	15536	1140	+	3
tRNA-Thr	15540	15613	74	+	1
tRNA-Pro	15615	15684	70	-	2
D-Loop	15687	>16711	>955	+	0

Supplementary Table S3. Composition and skewness of protein-coding genes (PCGs), tRNAs, rRNAs, and control region of *Trachemys* species and related taxa.

Taxon	Accession no.	A (%)	C (%)	G (%)	T (%)	GC (%)	AT skew	GC skew
**Complete mitogenome**								
Graptemys ouachitensis	OP115973	34.1	26.0	13.1	26.8	38.6	0.120	-0.330
Pseudemys concinna	OM935747	34.4	26.1	13.0	26.6	38.6	0.128	-0.340
Trachemys medemi	OZ183365	34.3	26.1	12.8	26.6	39.0	0.123	-0.342
Trachemys scripta elegans	KM216748	34.1	26.0	12.9	26.9	38.7	0.118	-0.337
Trachemys scripta scripta	KM216749	34.2	26.0	12.9	26.9	38.7	0.120	-0.337
Trachemys scripta troostii	MW122292	34.2	26.0	12.9	26.9	38.7	0.120	-0.337
Trachemys decorata	OX453475	34.1	26.2	12.9	26.7	38.6	0.122	-0.340
Trachemys decussata angusta	OX453476	34.3	26.2	12.7	26.8	38.5	0.123	-0.347
Trachemys decussata decussata	OX453477	34.3	26.2	12.8	26.7	38.5	0.125	-0.344
Trachemys grayi emolli	OX453490	34.3	26.1	12.8	26.8	38.9	0.123	-0.342
Trachemys grayi panamensis	OX453491	34.3	26.1	12.8	26.8	38.9	0.123	-0.342
Trachemys stejnegeri vicina	OX453495	34.2	26.2	12.9	26.8	38.6	0.121	-0.340
Trachemys terrapen	OX453498	34.3	26.2	12.8	26.6	38.5	0.126	-0.344
Trachemys venusta venusta	OX453499	34.0	26.0	12.9	26.8	38.5	0.118	-0.337
Trachemys venusta taylori	OZ038157	34.0	26.0	12.9	26.8	38.5	0.118	-0.337
Trachemys venusta cataspila	OZ038159	34.8	26.2	13.0	26.8	38.9	0.130	-0.337
Trachemys venusta uhrigi	OZ038162	34.1	26.1	12.9	26.9	38.8	0.118	-0.339
**PCGs**								
Graptemys ouachitensis	OP115973	33.2	27.6	11.8	27.3	39.4	0.098	-0.401
Pseudemys concinna	OM935747	33.6	27.7	11.7	27.0	39.4	0.109	-0.406
Trachemys medemi	OZ183365	33.4	27.9	11.7	27.1	39.5	0.104	-0.409
Trachemys scripta elegans	KM216748	33.4	27.5	11.6	27.4	39.1	0.099	-0.407
Trachemys scripta scripta	KM216749	33.4	27.5	11.6	27.4	39.1	0.099	-0.407
Trachemys scripta troostii	MW122292	33.5	27.5	11.6	27.4	39.1	0.100	-0.407
Trachemys decorata	OX453475	33.4	27.8	11.6	27.3	39.3	0.101	-0.411
Trachemys decussata angusta	OX453476	33.4	27.9	11.5	27.2	39.3	0.102	-0.416
Trachemys decussata decussata	OX453477	33.4	27.8	11.6	27.2	39.3	0.102	-0.411
Trachemys grayi emolli	OX453490	33.6	27.8	11.5	27.2	39.2	0.105	-0.415
Trachemys grayi panamensis	OX453491	33.6	27.7	11.5	27.2	39.2	0.105	-0.413
Trachemys stejnegeri vicina	OX453495	33.3	27.7	11.6	27.3	39.3	0.099	-0.410
Trachemys terrapen	OX453498	33.4	27.8	11.6	27.3	39.3	0.101	-0.411
Trachemys venusta venusta	OX453499	33.2	27.7	11.8	27.3	39.4	0.098	-0.403
Trachemys venusta taylori	OZ038157	33.1	27.7	11.9	27.3	39.5	0.096	-0.400
Trachemys venusta cataspila	OZ038159	33.2	27.7	11.9	27.3	39.5	0.098	-0.400
Trachemys venusta uhrigi	OZ038162	33.2	27.7	11.8	27.3	39.4	0.098	-0.403
**tRNAs**								
Graptemys ouachitensis	OP115973	34.3	22.5	16.0	27.2	38.2	0.116	-0.169

Taxon	Accession no.	A (%)	C (%)	G (%)	T (%)	GC (%)	AT skew	GC skew
**tRNAs (continued)**								
Pseudemys concinna	OM935747	34.6	22.6	15.7	27.1	38.1	0.122	-0.180
Trachemys medemi	OZ183365	34.8	22.4	15.8	26.9	38.1	0.128	-0.173
Trachemys scripta elegans	KM216748	34.9	22.5	15.6	27.0	37.9	0.128	-0.181
Trachemys scripta scripta	KM216749	34.9	22.5	15.6	27.0	37.9	0.128	-0.181
Trachemys scripta troostii	MW122292	34.7	22.6	15.7	27.1	38.1	0.123	-0.180
Trachemys decorata	OX453475	34.7	22.6	15.7	27.1	38.1	0.123	-0.180
Trachemys decussata angusta	OX453476	34.7	22.6	15.6	27.1	38.0	0.123	-0.183
Trachemys decussata decussata	OX453477	34.7	22.8	15.5	27.0	38.1	0.125	-0.191
Trachemys grayi emolli	OX453490	34.4	22.2	15.5	27.9	37.7	0.104	-0.178
Trachemys grayi panamensis	OX453491	34.3	22.3	15.6	27.9	37.8	0.103	-0.177
Trachemys stejnegeri vicina	OX453495	34.7	22.8	15.6	26.9	38.2	0.127	-0.188
Trachemys terrapen	OX453498	34.9	22.8	15.4	26.9	38.0	0.130	-0.194
Trachemys venusta venusta	OX453499	34.6	22.7	15.7	27.0	38.2	0.123	-0.182
Trachemys venusta taylori	OZ038157	34.6	22.7	15.7	27.0	38.2	0.123	-0.182
Trachemys venusta cataspila	OZ038159	34.7	22.7	15.7	27.0	38.1	0.125	-0.182
Trachemys venusta uhrigi	OZ038162	34.6	22.6	15.7	27.0	38.1	0.123	-0.180
**rRNAs**								
Graptemys ouachitensis	OP115973	38.7	22.5	16.7	22.1	38.8	0.273	-0.148
Pseudemys concinna	OM935747	38.8	22.7	16.5	21.9	39.0	0.278	-0.158
Trachemys medemi	OZ183365	38.6	23.1	16.7	21.6	39.4	0.282	-0.161
Trachemys scripta elegans	KM216748	38.5	22.9	16.7	21.9	39.3	0.275	-0.157
Trachemys scripta scripta	KM216749	38.5	22.9	16.8	21.8	39.2	0.277	-0.154
Trachemys scripta troostii	MW122292	38.5	22.9	16.7	21.9	39.3	0.275	-0.157
Trachemys decorata	OX453475	38.7	23.3	16.7	21.3	39.7	0.290	-0.165
Trachemys decussata angusta	OX453476	38.7	23.3	16.5	21.5	39.5	0.286	-0.171
Trachemys decussata decussata	OX453477	38.7	23.1	16.6	21.6	39.4	0.284	-0.164
Trachemys grayi emolli	OX453490	38.9	22.9	16.3	21.9	39.3	0.280	-0.169
Trachemys grayi panamensis	OX453491	38.9	23	16.4	21.7	39.4	0.284	-0.168
Trachemys stejnegeri vicina	OX453495	38.8	23	16.7	21.5	39.4	0.287	-0.159
Trachemys terrapen	OX453498	38.8	23.2	16.6	21.3	39.5	0.291	-0.166
Trachemys venusta venusta	OX453499	38.6	22.9	16.5	22.0	39.0	0.274	-0.162
Trachemys venusta taylori	OZ038157	38.8	22.9	16.4	21.9	38.9	0.278	-0.165
Trachemys venusta cataspila	OZ038159	38.7	22.9	16.4	22.0	38.8	0.275	-0.165
Trachemys venusta uhrigi	OZ038162	38.7	22.9	16.4	22.0	38.9	0.275	-0.165
**Control Region**								
Graptemys ouachitensis	OP115973	31.5	21.9	13.3	33.4	31.2	-0.029	-0.244
Pseudemys concinna	OM935747	33.2	20.9	12.8	33.1	29.9	0.002	-0.240
Trachemys medemi	OZ183365	33.7	20.7	12.0	33.5	32.7	0.002	-0.266
Trachemys scripta elegans	KM216748	30.2	22.1	14.0	33.7	34.9	-0.055	-0.224

Taxon	Accession no.	A (%)	C (%)	G (%)	T (%)	GC (%)	AT skew	GC skew
**Control Region (continued)**								
Trachemys scripta scripta	KM216749	30.4	22.1	13.9	33.5	34.7	-0.049	-0.228
Trachemys scripta troostii	MW122292	30.4	22.2	14	33.4	34.9	-0.047	-0.227
Trachemys decorata	OX453475	30.3	21.1	13.8	34.8	30.5	-0.069	-0.209
Trachemys decussata angusta	OX453476	31.5	20.6	12.9	35.0	29.5	-0.053	-0.230
Trachemys decussata decussata	OX453477	31.5	21.2	13.1	34.2	30.0	-0.041	-0.236
Trachemys grayi emolli	OX453490	30.4	21.3	14.0	34.3	30.9	-0.060	-0.207
Trachemys grayi panamensis	OX453491	30.1	20.9	14.2	34.7	30.8	-0.071	-0.191
Trachemys stejnegeri vicina	OX453495	30.2	21.2	13.7	35.0	30.5	-0.074	-0.215
Trachemys terrapen	OX453498	32.5	21.1	12.5	33.8	33.7	-0.020	-0.256
Trachemys venusta venusta	OX453499	32.2	21.2	12.8	33.8	30.3	-0.024	-0.247
Trachemys venusta taylori	OZ038157	32.4	21.4	12.6	33.6	30.3	-0.018	-0.259
Trachemys venusta cataspila	OZ038159	31.6	22.2	12.9	33.3	34.2	-0.026	-0.265
Trachemys venusta uhrigi	OZ038162	31.8	22.2	12.7	33.3	34.1	-0.023	-0.272

Supplementary Table S4. Data blocks of the mtDNA alignment used for phylogenetic analyses.

tRNA-Phe	1-72
12S	73-1049
tRNA-Val	1050-1121
16S	1122-2757
tRNA-Leu	2758-2833
ND1_pos1	2834-3799\3
ND1_pos2	2835-3799\3
ND1_pos3	2836-3799\3
tRNA-Ile-Gln	3800-4006
ND2_pos1	4007-5044\3
ND2_pos2	4008-5044\3
ND2_pos3	4009-5044\3
tRNA-Trp-Tyr	5045-5400
CO1_pos1	5401-6942\3
CO1_pos2	5402-6942\3
CO1_pos3	5403-6942\3
tRNA-Ser-Asp	6943-7078
CO2_pos1	7079-7762\3
CO2_pos2	7080-7762\3
CO2_pos3	7081-7762\3
tRNA-Lys	7763-7836
ATP8_pos1	7837-7992\3
ATP8_pos2	7838-7992\3
ATP8_pos3	7839-7992\3
ATP6_pos1	7993-8640\3
ATP6_pos2	7994-8640\3
ATP6_pos3	7995-8640\3
CO3_pos1	8641-9420\3
CO3_pos2	8642-9420\3
CO3_pos3	8643-9420\3
tRNA-Gly	9421-9489
ND3_pos1	9490-9838\3
ND3_pos2	9491-9838\3
ND3_pos3	9492-9838\3
tRNA-Arg	9839-9908
ND4L_pos1	9909-10197\3
ND4L_pos2	9910-10197\3
ND4L_pos3	9911-10197\3
ND4_pos1	10198-11568\3
ND4_pos2	10199-11568\3
ND4_pos3	10200-11568\3
tRNA-His-Leu	11569-11776
ND5_pos1	11777-13615\3
ND5_pos2	11778-13615\3
ND5_pos3	11779-13615\3
ND6_pos1	13616-14137\3
ND6_pos2	13617-14137\3
ND6_pos3	13618-14137\3
tRNA-Glu	14138-14205
CYTB-pos1	14206-15342\3
CYTB-pos2	14207-15342\3
CYTB-pos3	14208-15342\3
tRNA-Thr-Pro	15343-15486
Control	15487-16562

Supplementary Table S5. Best evolutionary models and partitioning schemes determined by PartitionFinder 2 for the complete mitogenome dataset. Estimated with the greedy search scheme and the Bayesian Information Criterion.

Subset		
1	HKY+I+G	Control, tRNA-Glu, ND5_pos1, ND5_pos2, ND4L_pos1, ND2_pos2, ND4_pos1, ATP6_pos2, CYTB_pos2, CO1_pos2, ND1_pos2, CO3_pos1, tRNA-Lys, CO3_pos2, tRNA-Val, tRNA-Phe, tRNA-Leu, ND3_pos2, CO1_pos1, ND1_pos1, ND6_pos1_tRNA-Thr-Pro, ND4L_pos2, ATP8_pos1, ND3_pos1, CYTB_pos1, 16S, ATP6_pos1, ND4_pos2, ND2_pos1, ATP8_pos2, ATP8_pos3, CO2_pos2, tRNA-Arg, tRNA-Gly, tRNA-His-Leu, tRNA-Trp-Tyr, tRNA-Arg, tRNA-Ile-Gln, CO2_pos1, tRNA-Thr-Pro, 12S, ND6_pos2
2	GTR+G	CYTB_pos3, ND2_pos3, ND5_pos3, ND6_pos3, ND4L_pos3, CO1_pos3, ND3_pos3, ND1_pos3, ATP6_pos3, ND4_pos3, CO2_pos3, CO3_pos3

References

Edgar RC (2004) MUSCLE: Multiple sequence alignment with high accuracy and high throughput. Nucleic Acids Research 32: 1792–1797. https://doi.org/10.1093/nar/gkh340

Fritz U, Stuckas H, Vargas-Ramírez M, Hundsdörfer AK, Maran J, Päckert M (2012) Molecular phylogeny of Central and South American slider turtles: Implications for biogeography and systematics (Testudines: Emydidae: *Trachemys*). Journal of Zoological Systematics and Evolutionary Research 50: 125–136. https://doi.org/10.1111/j.1439-0469.2011.00647.x

Fritz U, Kehlmaier C, Scott RJ, Fournier R, McCranie JR, Gallego-García N (2023) Central American *Trachemys* revisited: New sampling questions current understanding of taxonomy and distribution (Testudines: Emydidae). Vertebrate Zoology 73: 513–523. https://doi.org/ 10.3897/vz.73.e104438

Fritz U, Herrmann HW, Rosen PC, Auer M, Vargas-Ramírez M, Kehlmaier C (2024) *Trachemys* in Mexico and beyond: Beautiful turtles, taxonomic nightmare, and a mitochondrial poltergeist (Testudines: Emydidae). Vertebrate Zoology 74: 435–452. https://doi.org/10.3897/vz.74.e125958

Lanfear R, Frandsen PB, Wright AM, Senfeld T, Calcott B (2017) PartitionFinder 2: New methods for selecting partitioned models of evolution for molecular and morphological phylogenetic analyses. Molecular Biology and Evolution 34: 772–773. https://doi.org/10.1093/molbev/msw260

Nguyen LT, Schmidt HA, von Haeseler A, Mihn BQ (2015) IQ-TREE: A fast and effective stochastic algorithm for estimating maximum-likelihood phylogenies. Molecular Biology and Evolution 32: 268–274. https://doi.org/10.1093/molbev/msu300

Park J, Cheon S, Park SM, Sung HC, Lee DH (2022) Complete mitochondrial genome of the river cooter (*Pseudemys concinna*, Testudines: Emydidae) in Korea. Mitochondrial DNA B 7: 1659–1661. https://doi.org/10.1080/23802359.2022.2119820

Park J, Park SM, Moon JI, Song YJ, Choi JH, Sung HC, Lee DH (2021) Complete mitochondrial genome of the distinct red-eared slider (*Trachemys scripta* ssp., Testudines: Emydidae) in Korea. Mitochondrial DNA B 6: 1077–1079. https://doi.org/10.1080/23802359.2021.1899866

Rambaut A, Drummond AJ, Xie D, Baele G, Suchard MA (2018) Posterior summarization in Bayesian phylogenetics using Tracer 1.7. Systematic Biology 67: 901–904. https://doi.org/10.1093/sysbio/syy032

Ronquist F, Teslenko M, van der Mark P, Ayres DL, Darling A, Höhna S, Larget B, Liu L, Suchard MA, Huelsenbeck JP (2012) MrBayes 3.2: Efficient Bayesian phylogenetic inference and model choice across a large model space. Systematic Biology 61: 539–542. https://doi.org/10.1093/sysbio/sys029

Russell RD, Beckenbach AT (2008) Recoding of translation in turtle mitochondrial genomes: programmed frameshift mutations and evidence of a modified genetic code. Journal of Molecular Evolution 67: 682–695. https://doi.org/10.1007/s00239-008-9179-0

Ryu G, Moon J-I, Song Y-J, Park J, Park S-M, Choi JH, Sung H-C, Lee D-H (2021) Complete mitochondrial genome of the Cumberland slider (*Trachemys scripta troostii*, Testudines: Emydidae) in Korea. Mitochondrial DNA B 6: 1131–1133. https://doi.org/10.1080/23802359.2021.1902410.

Yu D, Fang X, Storey KB, Zhang Y, Zhang J (2016) Complete mitochondrial genomes of the yellow-bellied slider turtle *Trachemys scripta scripta* and anoxia tolerant red-eared slider *Trachemys scripta elegans*. Mitochondrial DNA A 27: 2276–2277. https://doi.org/10.3109/19401736.2014.984178
